# Supplementary material for: Placental Streptococcus agalactiae DNA is associated with neonatal unit admission and foetal pro-inflammatory cytokines in term infants
Source: Nat Microbiol. 2023 Nov 29;8(12):2338–48. doi: 10.1038/s41564-023-01528-2 (PMC10686823; doi:10.1038/s41564-023-01528-2)
Supplement: Supplementary file 1 — Supplementary Tables 1–9. [file 41564_2023_1528_MOESM1_ESM.pdf]

nature microbiology

Article

https://doi.org/10.1038/s41564-023-01528-2

Placental *Streptococcus agalactiae* DNA is associated with neonatal unit admission and foetal pro-inflammatory cytokines in term infants

In the format provided by the authors and unedited

Supplementary Tables

Contents.

Supplementary Table 1. Characteristics of cases and controls from discovery study (n=436).

Supplementary Table 2. Characteristics of cases and controls from validation study (n=925).

Supplementary Table 3. Ordinal scale for the clinical classification of neonatal unit admission in relation to evidence supporting a diagnosis of sepsis.

Supplementary Table 4. Weekly GBS positive and total deliveries in the validation study.

Supplementary Table 5. Association between mode of delivery and placental GBS.

Supplementary Table 6. Association between HVS (GBS positive versus negative swab) and the risk of neonatal unit admission, classified by clinical assessment or histopathology.

Supplementary Table 7. Distribution of samples by case/control-status and GBS-status in the qPCR plates.

Supplementary Table 8. Association between the presence of GBS DNA and RNA in the placenta and the risk of neonatal unit admission, classified by clinical assessment or histopathology.

Supplementary Table 9. Specificity of the PCR-qPCR 16S assay.

References.

Supplementary Table 1. Characteristics of cases and controls from discovery study (n=436).

Characteristic	Cases (n=41)	Controls (n=395)
**Age, years**	31 (28 to 34)	30 (27 to 34)
**Age stopped FTE, years**	21 (17 to 23)	21 (18 to 23)
Missing	2 (5)	12 (3)
**BMI, kg/m^2**	24 (23 to 28)	25 (23 to 28)
**Smoker**	6 (15)	25 (6)
**Any alcohol consumption**	0 (0)	18 (5)
**Deprivation score quartile**		
1 (lowest)	17 (41)	100 (25)
2	7 (17)	78 (20)
3	9 (22)	101 (26)
4 (highest)	7 (17)	88 (22)
Missing	1 (2)	28 (7)
**Ethnicity**		
Non white	3 (7)	18 (5)
White	38 (93)	369 (93)
Missing	0 (0)	8 (2)
**Married**	28 (68)	271 (69)
**Induction of labor**	19 (46)	137 (35)
**Mode of delivery**		
Unassisted vaginal	12 (29)	174 (44)
Assisted vaginal	11 (27)	75 (19)
Intrapartum CS	9 (22)	74 (19)
Pre-labor CS	9 (22)	72 (18)
**Gestational age at delivery, weeks**	39.1 (38.0 to 40.3)	40.1 (39.3 to 41.1)
**Birth weight, g**	2750 (2375 to 3205)	3375 (2910 to 3660)

Data are expressed as median (inter-quartile range) or n (%), as appropriate. For fields where there is no category labelled 'missing', data were 100% complete. Maternal age was defined as age at recruitment. All other maternal characteristics were defined by self-report at the 20 weeks questionnaire, from examination of the clinical case record, or linkage to the hospital's electronic databases. Socio-economic status was quantified using the Index of Multiple Deprivation (IMD) 2007, which is based on census data from the area of the mother's postcode, and classified into study specific quartiles. FTE denotes full time education; BMI denotes body mass index; CS denotes Caesarean section.

Supplementary Table 2. Characteristics of cases and controls from validation study (n=925).

Characteristic	Cases (n=239)	Controls (n=686)
**Age, years**	31 (27 to 35)	30 (27 to 33)
**Age stopped FTE, years**	21 (18 to 23)	21 (18 to 23)
Missing	8 (3)	22 (3)
**BMI, kg/m^2**	25 (22 to 28)	24 (22 to 27)
**Smoker**	11 (5)	32 (5)
**Any alcohol consumption**	13 (5)	30 (4)
Missing	0 (0)	1 (<1)
**Deprivation score quartile**		
1 (lowest)	45 (19)	157 (23)
2	65 (27)	160 (23)
3	65 (27)	157 (23)
4 (highest)	55 (23)	182 (27)
Missing	9 (4)	30 (4)
**Ethnicity**		
Non white	15 (6)	45 (7)
White	223 (93)	628 (92)
Missing	1 (<1)	13 (2)
**Married**	165 (69)	476 (69)
**Induction of labor**	108 (45)	204 (30)
**Mode of delivery**		
Unassisted vaginal	77 (32)	361 (53)
Assisted vaginal	65 (27)	154 (22)
Intrapartum CS	81 (34)	109 (16)
Pre-labor CS	16 (7)	61 (9)
Missing	0 (0)	1 (<1)
**Gestational age at delivery, weeks**	40.4 (39.0 to 41.4)	40.4 (39.4 to 41.1)
**Birth weight, g**	3490 (3180 to 3840)	3435 (3155 to 3740)

Data are expressed as median (inter-quartile range) or n (%), as appropriate. For fields where there is no category labelled 'missing', data were 100% complete. Maternal age was defined as age at recruitment. All other maternal characteristics were defined by self-report at the 20 weeks questionnaire, from examination of the clinical case record, or linkage to the hospital's electronic databases. Socio-economic status was quantified using the Index of Multiple Deprivation (IMD) 2007, which is based on census data from the area of the mother's postcode, and classified into study specific quartiles. FTE denotes full time education; BMI denotes body mass index; CS denotes Caesarean section.

Supplementary Table 3. Ordinal scale for the clinical classification of neonatal unit admission in relation to evidence supporting a diagnosis of sepsis.

Category	Definition
No sepsis	No clinical evidence of sepsis and septic screen and investigations not warranted
Possible sepsis	Clinical signs suggestive of sepsis to warrant sepsis screen (blood culture, FBC, CRP, Chest and abdominal X-rays if clinically indicated) and prophylactic antibiotics. Negative sepsis screen with no laboratory or radiological evidence of infection.
Probable but culture negative sepsis	Clinical signs AND laboratory and/or radiological evidence to warrant completion of a full course of antibiotics but culture negative
Proven GBS sepsis	Organism cultured from a normally sterile site

FBC denotes full blood count; CRP denotes C-reactive protein test. Laboratory evidence for probable sepsis were leucocytosis (white blood cell [WBC] count > 20 x 10^9/L), leucopenia (WBC count < 5 x 10^9/L), thrombocytopenia (platelet count < 150 x 10^9/L) and/or CRP > 10 mg/L. Radiological evidence for probable sepsis were presence of pulmonary infiltrates, consolidation and/or intestinal dilatation.

Supplementary Table 4. Weekly GBS positive and total deliveries in the validation study.

Delivery week	GBS positives (n)	Weekly deliveries (n)
2009w12	1	3
2009w14	1	5
2009w42	2	4
2010w4	1	2
2010w10	1	2
2010w12	1	8
2010w16	1	7
2010w27	1	3
2010w39	1	5
2011w17	1	3
2011w22	1	10
2011w25	1	7
2011w27	1	8
2011w28	1	4
2011w29	1	6
2011w35	1	5
2011w38	1	3
2011w45	1	5
2011w48	1	3
2011w51	1	6
2011w52	1	5
2012w2	1	12
2012w6	1	7
2012w13	1	7
2012w15	1	12
2012w19	1	4
2012w20	1	5
2012w24	1	3
2012w25	2	6
2012w37	1	3
2012w39	1	5
2012w52	1	5
2013w1	1	8
2013w4	1	5

n numbers of GBS positive and total deliveries per week in the validation study. The delivery week is indicated with the year first, followed by the week number. The temporal distribution of the GBS positive cases (n=36) is represented in Extended Data Fig. 4.

Supplementary Table 5. Association between mode of delivery and placental GBS.

	Placental GBS		
	No	Yes	Total
**Mode of delivery**			
Vaginal	638 (72)	19 (53)	657 (71)
C-section	250 (28)	17 (47)	267 (29)
Total	888 (100)	36 (100)	924 (100)

Data are expressed as n (%). The proportion of C-sections was higher among patients with placental GBS DNA (47% vs. 28%; OR=2.3 [95% CI = 1.2-4.4]; Pearson's X^2 test p=0.01). Mode of delivery was unknown for 1 patient of the validation study.

Supplementary Table 6. Association between HVS (GBS positive versus negative swab) and the risk of neonatal unit admission, classified by clinical assessment or histopathology.

	HVS		Univariable analysis			Multivariable analysis		
	Positive n (%)	Negative n (%)	OR	95% CI	p	OR	95% CI	p
**Neonatal unit admission**								
Not admitted (N=348)	36 (10.3)	312 (89.7)						
All admissions (N=119)	21 (17.6)	98 (82.4)	1.9	1.0 to 3.4	0.04	1.8	1.0 to 3.3	0.05
**By diagnosis**								
No sepsis (N=26)	4 (15.4)	22 (84.6)	1.6	0.6 to 4.8	0.42	1.6	0.5 to 5.0	0.42
Possible sepsis (N=50)	10 (20.0)	40 (80.0)	2.2	1.0 to 4.6	0.05	2.1	1.0 to 4.7	0.06
Probable sepsis (N=36)	5 (13.9)	31 (86.1)	1.4	0.6 to 3.6	0.51	1.3	0.5 to 3.7	0.59
Proven GBS sepsis (N=1)	1 (100.0)	0 (0.0)	N/A	N/A	0.11			
**By histopathology**								
No inflammation (N=89)	14 (15.7)	75 (84.3)	1.6	0.8 to 3.1	0.15	1.6	0.8 to 3.1	0.18
Chorioamnionitis (N=28)	7 (25.0)	21 (75.0)	2.9	1.1 to 6.9	0.02	3.2	1.2 to 8.4	0.02
Funisitis (N=17)	6 (35.3)	11 (64.7)	4.7	1.6 to 14.0	0.002	4.9	1.6 to 14.8	0.005

For univariable analysis, unadjusted odds ratio (OR) with Baptista-Pike mid-p 95% confidence interval (CI) and chi-square test p value are presented. Due to small numbers, Fisher's exact test two-sided P value is given for proven GBS sepsis. For multivariable analysis, OR, 95% CI and p values were estimated using logistic regression analysis adjusted for maternal characteristics (age, body mass index, smoking and marital status). Due to small numbers, multivariable analysis is omitted for proven GBS sepsis. Chorioamnionitis and funisitis were both present in 6 positive and in 9 negative HVS. The diagnosis for six cases of neonatal unit admission could not be confirmed due to missing information. HVS denotes high vaginal swab.

Supplementary Table 7. Distribution of samples by case/control-status and GBS-status in the qPCR plates.

Plate	Controls<br>Cases	GBS positives
1	50 (77)	2
	15 (23)	2
2	40 (74)	0
	14 (26)	1
3	57 (76)	0
	18 (24)	1
4	60 (80)	2
	15 (20)	0
5a	27 (73)	3
	10 (27)	2
5b & 9a	55 (72)	0
	21 (28)	0
6	53 (70)	1
	23 (30)	3
7	58 (79)	2
	15 (21)	1
8	49 (71)	2
	20 (29)	0
9b	27 (75)	2
	9 (25)	0
10	52 (74)	1
	18 (26)	2
11	58 (70)	2
	25 (30)	2
12	58 (74)	2
	20 (26)	1
13	42 (72)	1
	16 (28)	1
tot	925 (100)	36

Cases and controls of the validation study are defined as described in the Methods and expressed as n (%) in each qPCR plate. GBS positive samples are expressed as n in each qPCR plate.

Supplementary Table 8. Association between the presence of GBS DNA and RNA in the placenta and the risk of neonatal unit admission, classified by clinical assessment or histopathology.

	Placental GBS		Univariable analysis			Multivariable analysis		
	Positive n (%)	Negative n (%)	OR	95% CI	p	OR	95% CI	p
**Neonatal unit admission**								
Not admitted (N=681)	16 (2.3)	665 (97.7)						
All admissions (N=234)	13 (5.6)	221 (94.4)	2.4	1.2 to 5.1	0.02	2.3	1.1 to 4.9	0.03
**By diagnosis**								
No sepsis (N=58)	0 (0.0)	58 (100.0)						
Possible sepsis (N=88)	2 (2.3)	86 (97.7)	1.0	0.2 to 4.0	0.96	0.9	0.2 to 4.1	0.92
Probable sepsis (N=79)	9 (11.4)	70 (88.6)	5.3	2.2 to 11.9	<0.0001	5.1	2.1 to 12.0	0.0002
Proven GBS sepsis (N=3)	2 (66.7)	1 (33.3)	83.1	9.0 to 1201.9	0.002			
**By histopathology**								
No inflammation (N=181)	6 (3.3)	175 (96.7)	1.4	0.6 to 3.6	0.46	1.2	0.4 to 3.1	0.75
Chorioamnionitis (N=44)	6 (13.6)	38 (86.4)	6.6	2.5 to 18.0	<0.0001	7.1	2.6 to 19.6	0.0001
Funisitis (N=30)	5 (16.7)	25 (83.3)	8.3	3.1 to 23.5	<0.0001	7.9	2.6 to 24.1	0.0003

For univariable analysis, unadjusted odds ratio (OR) with Baptista-Pike mid-p 95% confidence interval (CI) and chi-square test p value are presented. Due to small numbers, Fisher's exact test two-sided P value is given for proven GBS sepsis. For multivariable analysis, OR, 95% CI and p values were estimated using logistic regression analysis adjusted for maternal characteristics (age, body mass index, smoking and marital status). Due to small numbers, univariable and multivariable analyses are omitted for no sepsis and multivariable analysis is omitted for proven GBS sepsis. Chorioamnionitis and funisitis were both present

in 4 cases where the placenta was GBS positive and in 19 of the cases where it was negative. The diagnosis for six cases of neonatal unit admission could not be confirmed due to missing information.

Supplementary Table 9. Specificity of the PCR-qPCR 16S assay.

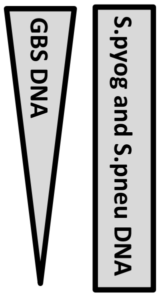

	GBS - *Streptococcus agalactiae* DNA (genomes/well)	*Streptococcus pneumoniae* DNA (genomes/well)	*Streptococcus pyogenes* DNA (genomes/well)	**qPCR**	
				**sip (Ct)**	**16S (Ct)**
**mix 1**	**90**	**900**	**900**	**30.3**	**27.3**
**mix 2**	**9**	**900**	**900**	**31.4**	**28.4**
**mix 3**	**0**	**900**	**900**	undet	undet

Bacterial DNA mixes 1, 2 or 3 contained 900 genome copies/well of *Streptococcus pyogenes*, 900 genome copies/well *Streptococcus pneumoniae* and 90, 9, 0 genome copies/well of GBS (*Streptococcus agalactiae*), respectively. Each mix was tested in triplicate using the qPCR assays targeting the *sip* and *16S* GBS genes. Undet, undetectable.
