# Supplementary material for: Placental Streptococcus agalactiae DNA is associated with neonatal unit admission and foetal pro-inflammatory cytokines in term infants
Source: Nat Microbiol. 2023 Nov 29;8(12):2338–48. doi: 10.1038/s41564-023-01528-2 (PMC10686823; doi:10.1038/s41564-023-01528-2)
Supplement: Supplementary file 2 — Reporting Summary [file 41564_2023_1528_MOESM2_ESM.pdf]

nature portfolio

Corresponding author(s): D. Stephen Charnock-Jones and Gordon C. S. Smith

Last updated by author(s): Sep 25, 2023

Reporting Summary

Nature Portfolio wishes to improve the reproducibility of the work that we publish. This form provides structure for consistency and transparency in reporting. For further information on Nature Portfolio policies, see our Editorial Policies and the Editorial Policy Checklist.

Statistics

For all statistical analyses, confirm that the following items are present in the figure legend, table legend, main text, or Methods section.

n/a	Confirmed	
☐	☒	The exact sample size (*n*) for each experimental group/condition, given as a discrete number and unit of measurement
☐	☒	A statement on whether measurements were taken from distinct samples or whether the same sample was measured repeatedly
☐	☒	The statistical test(s) used AND whether they are one- or two-sided<br>*Only common tests should be described solely by name; describe more complex techniques in the Methods section.*
☐	☒	A description of all covariates tested
☐	☒	A description of any assumptions or corrections, such as tests of normality and adjustment for multiple comparisons
☐	☒	A full description of the statistical parameters including central tendency (e.g. means) or other basic estimates (e.g. regression coefficient) AND variation (e.g. standard deviation) or associated estimates of uncertainty (e.g. confidence intervals)
☐	☒	For null hypothesis testing, the test statistic (e.g. *F*, *t*, *r*) with confidence intervals, effect sizes, degrees of freedom and *P* value noted<br>*Give P values as exact values whenever suitable.*
☒	☐	For Bayesian analysis, information on the choice of priors and Markov chain Monte Carlo settings
☒	☐	For hierarchical and complex designs, identification of the appropriate level for tests and full reporting of outcomes
☐	☒	Estimates of effect sizes (e.g. Cohen's *d*, Pearson's *r*), indicating how they were calculated

Our web collection on statistics for biologists contains articles on many of the points above.

Software and code

Policy information about availability of computer code

Data collection	The software used to collect data were the quantitative PCR machine software (QuantStudio 6 Flex system version 1.3, ThermoFisher Scientific) and the Ella platform software version 3.7.2.0 (Bio-Techne).
Data analysis	Statistical analyses were performed using Stata version 17 (StataCorp LLC) and GraphPad Prism version 9.2.0 (GraphPad Software LLC).

For manuscripts utilizing custom algorithms or software that are central to the research but not yet described in published literature, software must be made available to editors and reviewers. We strongly encourage code deposition in a community repository (e.g. GitHub). See the Nature Portfolio guidelines for submitting code & software for further information.

Data

Policy information about availability of data

All manuscripts must include a data availability statement. This statement should provide the following information, where applicable:

- Accession codes, unique identifiers, or web links for publicly available datasets
- A description of any restrictions on data availability
- For clinical datasets or third party data, please ensure that the statement adheres to our policy

Source data are provided with this paper. Any additional data are available from the corresponding authors at reasonable request and subject to a Data Transfer Agreement, as they include clinical information and in compliance with the ethical permission for the POP study. No participant identifiable information will be disclosed. Requests can be addressed to GCSS (gcss2@cam.ac.uk) and DSC-J (dscj1@cam.ac.uk) and will be answered within 1 month. Previously described

datasets5 are: the 16S rRNA gene sequencing datasets, which are publicly available under European Nucleotide Archive (ENA) accession number ERP109246; the metagenomics datasets, which are available with managed access in the European Genome-phenome Archive (EGA) accession number EGAD00001004198.

Human research participants

Policy information about studies involving human research participants and Sex and Gender in Research.

Reporting on sex and gender	The data reported in this study are related to samples from women of child-bearing age and their infants. The manuscript does not include any analysis based on the biological sex of the babies.
Population characteristics	Samples were from the Pregnancy Outcome Prediction (POP) study. In the whole POP study population (n=4212), the median age, height and BMI (IQR) were 30.3 (26.8 to 33.4) years, 165 (161 to 169) cm, 24.1 (21.8 to 27.3) kg/m2, respectively, and 13% of the women were smokers at recruitment. Detailed characteristics of women whose samples were selected for this study are given in Supplementary Tables 1 and 2. Participants received no compensations for being part of the study.<br>In the discovery study (n=436), the median maternal age varied between 31 and 30 years between the groups of 41 cases and 395 controls. The median BMI was similar between the groups (24kg/m2 in cases and 25kg/m2 in controls). The prevalence of smoking at booking was 15% in cases and 6% among the controls and the prevalence of alcohol consumption was 0% in cases and 5% among the controls.<br>In the validation study (n=925), the median maternal age varied between 31 and 30 years between the groups of 239 cases and 686 controls. The median BMI was similar between the groups (25kg/m2 in cases and 24kg/m2 in controls). The prevalence of smoking at booking was 5% in both groups and the prevalence of alcohol consumption was 5% in cases and 4% among the controls.
Recruitment	Samples were from the Pregnancy Outcome Prediction (POP) study. Nulliparous women with a viable singleton pregnancy who attended their dating ultrasound scan at the Rosie Hospital (Cambridge, UK) between 14 January 2008 and 31 July 2012 were eligible (n=8028). Of these, 4512 women (56%) provided an informed consent and were recruited. Participants received no compensations for being part of the study. The recruited and non-recruited women were broadly comparable, although according to the hospital record data the women who were recruited were slightly older, more often of white ethnic origin and less likely to smoke. Women were excluded because they delivered elsewhere (n=233) or withdrew their consent (n=67) (Supplementary Figure 1). The cohort of 4212 women used for the sample selection in the present study can be regarded as fairly well representative of the eligible population. See Sovio et al Lancet 2015 PMID 26360240 and Gaccioli et al Placenta 2017 PMCID PMC5701771 for a complete description.
Ethics oversight	The Pregnancy Outcome Prediction study was approved by the Cambridgeshire 2 Research Ethics Committee (reference number 07/H0308/163).

Note that full information on the approval of the study protocol must also be provided in the manuscript.

Field-specific reporting

Please select the one below that is the best fit for your research. If you are not sure, read the appropriate sections before making your selection.

☒ Life sciences ☐ Behavioural & social sciences ☐ Ecological, evolutionary & environmental sciences

For a reference copy of the document with all sections, see nature.com/documents/nr-reporting-summary-flat.pdf

Life sciences study design

All studies must disclose on these points even when the disclosure is negative.

Sample size	A power calculation was performed during the planning phase of the POP study and it is described in Pasupathy et al (BMC Pregnancy and Childbirth 2008 PMID 19019223). In brief, the sensitivity of different models for a given screen positive rate was quantified by 95% confidence intervals. The calculations indicated that the study was likely to provide reasonably precise estimates of sensitivity for conditions with a 3% incidence. The use of a nested case-control design with matching of cases and controls on key maternal characteristics was also planned in advance in the context of expensive or labor intensive methodologies (Pasupathy et al).<br><br>The sample size and power calculation for the discovery study have been previously described in detail (de Goffau et al Nature 2019 PMID 31367035).<br><br>The validation case-control study (239 cases and 686 controls) included all the eligible participants with a term pregnancy, available placental biopsies and a live born infant admitted to the neonatal unit (without limit of timing or duration). Controls were pregnancies where were the infant was not admitted to the neonatal unit and were selected in a ratio of two controls for each case. Power calculations were performed for all analyses using the available sample size to estimate the statistical power to detect a given strength of association based on alpha (two-sided) = 0.05. The power calculation for the primary outcome (admission of the infant to the neonatal unit) was based on observing the same relative risk and proportions of exposure and outcome in the validation study as observed in the development study. This analysis indicated that we had >99% power to replicate the original finding with the sample size of the validation study. Power calculations for all the secondary outcomes (fetal cytokine storm, probable and confirmed sepsis, chorioamnionitis and funisitis) were based on the observed numbers of GBS positive and GBS negative placentas in the validation study, the observed proportion of the given outcome in the GBS negative group of the validation study, and a range of possible odds ratios (see Supplementary Fig. 10).

Data exclusions	A total of 4512 women with a viable singleton pregnancy were recruited to the POP study. The only clinical exclusion criterion for the study was multiple pregnancy. In the current work preterm births were excluded.<br><br>Samples were excluded from the PCR/qPCR analysis if the RNaseP signal was not detectable, indicating absence of gDNA in the PCR assay or a technical failure.<br><br>In the primary analysis of inflammatory cytokines (Ella, Bio-Techne), positive samples which were weakly positive by 16S rRNA PCR-qPCR (i.e. GBS 16S rRNA amplicons >0% and <1%; n=5) were excluded.
Replication	The current work includes 2 non-overlpping cohorts: 1) a discovery case-control study where the initial observation was made, and 2) a validation case-control study where the findings were replicated.
Randomization	The POP study is a prospective cohort study of nulliparous women attending the Rosie Hospital (Cambridge, UK) for their dating ultrasound scan. All eligible participants were included. The current work excluded preterm births.<br><br>For the analyses and experiments performed in this manuscript, participants were allocated into groups based on pregnancy outcome (details in Methods). Outcome data were ascertained by review of each woman's paper case record by an Academic Clinical Fellow and a Neonatologist, and by record linkage to clinical electronic databases.<br>During the experiments, batches contain samples from both cases and controls.
Blinding	All the aspects of the POP study were conducted blind: the results of the research ultrasound scans and the biochemical marker data were not revealed to the clinicians, patients and researchers performing the downstream experiments. Classification of cases of NNU admission by evidence for sepsis and histopathological examination of the fetal membranes and the umbilical cord was all performed blind to placental GBS status. In order to evaluate the associations between the exposure (i.e. presence of GBS DNA in the placenta) and the studied clinical outcomes (infant NICU admission, fetal cytokine storm, probable and proven sepsis, chorioamnionitis and funisitis), data were unblinded at the statistical analysis stage.

Reporting for specific materials, systems and methods

We require information from authors about some types of materials, experimental systems and methods used in many studies. Here, indicate whether each material, system or method listed is relevant to your study. If you are not sure if a list item applies to your research, read the appropriate section before selecting a response.

Materials & experimental systems

n/a	Involved in the study
☒	☐ Antibodies
☒	☐ Eukaryotic cell lines
☒	☐ Palaeontology and archaeology
☒	☐ Animals and other organisms
☒	☐ Clinical data
☒	☐ Dual use research of concern

Methods

n/a	Involved in the study
☒	☐ ChIP-seq
☒	☐ Flow cytometry
☒	☐ MRI-based neuroimaging
